# Impact of B-Ring Substitution and Acylation with Hydroxy Cinnamic Acids on the Inhibition of Porcine α-Amylase by Anthocyanin-3-Glycosides

**DOI:** 10.3390/foods9030367

**Published:** 2020-03-21

**Authors:** Julia A. H. Kaeswurm, Lisa Könighofer, Melanie Hogg, Andreas Scharinger, Maria Buchweitz

**Affiliations:** 1 Institute of Biochemistry and Technical Biochemistry, Department of Food Chemistry, University of Stuttgart, 70569 Stuttgart, Germany; julia.kaeswurm@lc.uni-stuttgart.de (J.A.H.K.); lisa@freenet.de (L.K.); Melanie.Hogg@gmx.de (M.H.); 2 Chemisches und Veterinäruntersuchungsamt Karlsruhe, Weißenburger Str. 3, 76187 Karlsruhe, Germany; andreas.scharinger@cvuaka.bwl.de

**Keywords:** α-amylase, acylated anthocyanins, non-acylated anthocyanins, black carrot anthocyanins, inhibition kinetics, isothermal titration calorimetry (ITC)

## Abstract

An inhibitory effect on α-amylase and α-glucosidase is postulated for polyphenols. Thus, ingestion of those secondary plant metabolites might reduce postprandial blood glucose level (hyperglycemia), which is a major risk factor for diabetes mellitus type II. In addition to a previous study investigating structure−effect relationships of different phenolic structures, the effect of anthocyanins is studied in detail here, by applying an α-amylase activity assay, on the basis of the conversion of 2-chloro-4-nitrophenyl-4-O-ß-galactopyranosyl maltoside (GalG_2_CNP) and detection of CNP release by UV/Vis spectroscopy and isothermal titration calorimetry (ITC). All anthocyanin-3-glucosides showed a mixed inhibition with a strong competitive proportion, K_ic_ < 134 µM and K_iu_ < 270 µM; however, the impact of the B-ring substitution was not statistically significant. UV/Vis detection failed to examine the inhibitory effect of acylated cyanidins isolated from black carrot (*Daucus carota* ssp. *Sativus* var. *Autrorubens Alef.*). However, ITC measurements reveal a much stronger inhibitory effect compared to the cyanidin-3-glucoside. Our results support the hypothesis that anthocyanins are efficient α-amylase inhibitors and an additional acylation with a cinnamic acid boosts the observed effect. Therefore, an increased consumption of vegetables containing acylated anthocyanin derivatives might help to prevent hyperglycemia.

## 1. Introduction

**Figure 1:**
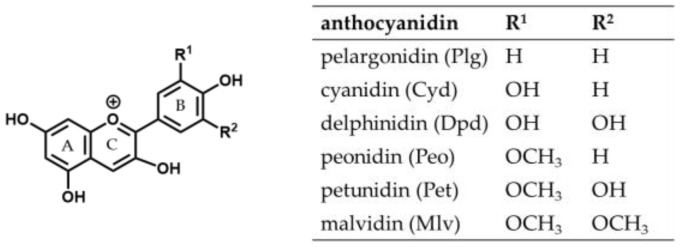
Structure of the six most frequently occurring anthocyanidins.

Anthocyanins are water-soluble natural red and blue pigments found in flowers, fruits, and vegetables. They are polyphenols and belong to the subgroup of flavonoids [1], consisting of an anthocyanidin aglycon to which a saccharide moiety is bound. There are many different anthocyanin structures in nature. They differ in the number and position of hydroxyl and methoxy groups on the aglycon and the nature and position of glycosyl residues [2]. Approximately 90% of all anthocyanins in plants possess a cyanidin (Cyd), delphinidin (Dpd), pelargonidin (Plg), petunidin (Pet), peonidin (Peo), or malvidin (Mlv) structure as the skeleton (Figure 1) [3].

Anthocyanins are often found in foods or coloring extracts added to foods for coloring purposes [2]. Particularly high anthocyanin contents in food have been found in berries and red cabbage. It is assumed that US citizens ingest approximately 12.5 mg of anthocyanins daily, of which 77% are non-acetylated and 23% are acetylated derivatives found in vegetables.

**Figure 2:**
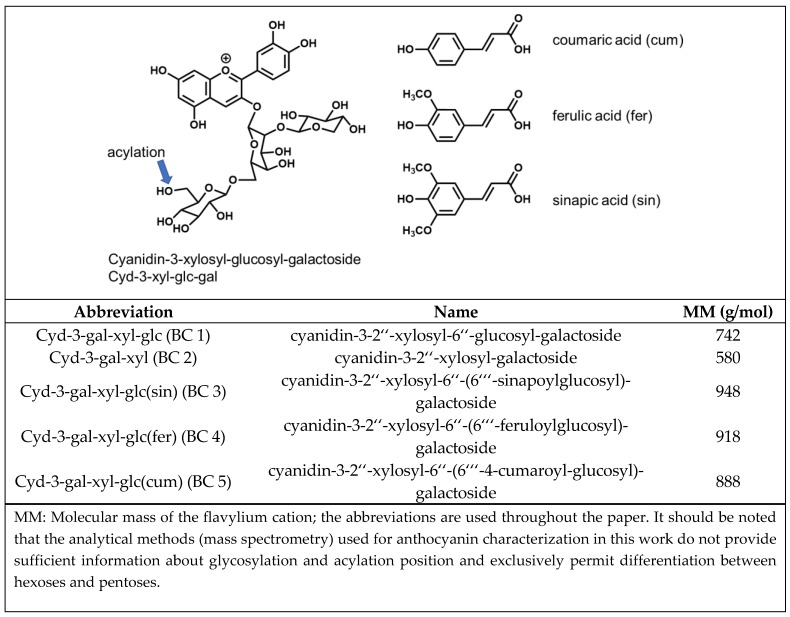
Anthocyanin structures isolated from black carrot according to Gläßgen et al. [7].

Due to its high anthocyanin content (4−1799 mg/100 g dry weight or 1−44 mg/100 g fresh weight [3], 1−98 mg/100 g fresh weight [4]), which corresponds to 25%−50% of the total polyphenol content [5], black carrot (*Daucus carota* ssp. *Sativus* var. *Autrorubens Alef.*) is a popular source for the preparation of coloring extracts. The five major anthocyanins of black carrot are cyanidin-based structures (Figure 2). These are cyanidin-3-xylosyl-galactoside (Cyd-gal-xyl), cyanidin-3-xylosyl-glucosyl galactoside (Cyd-gal-xyl-glc) and its derivatives acylated with sinapic acid (Cyd-gal-xyl-glc(sin)), ferulic acid (Cyd-gal-xyl-glc(fer)), and coumaric acid (Cyd-gal-xyl-glc(cum)) [5]. Peo- and Plg-glycosides have been detected as well, to a markedly lesser extent, however. Acylated structures usually make up 55%−99% of black carrot anthocyanins. The most abundant anthocyanin is Cyd-gal-xyl(fer), which accounts for 40%−80% of the total anthocyanin content [4,5]. Due to the high proportion of acylated anthocyanins, which are responsible for the good color stability under weakly acidic conditions, coloring extracts prepared from black carrot are frequently applied to dairy products [6].

Diabetes mellitus is a chronic disease that is becoming a serious global health problem [8]. Control of the postprandial blood glucose level is an effective method to prevent hyperglycemia and thus, the development of diabetes type II [9]. Acarbose (ACA) is a commercially available drug to inhibit enzymes of the carbohydrate metabolism and to reduce the absorption of glucose into the body [10], hence lowering postprandial glucose levels and preventing hyperglycemia. Inhibitory effects of anthocyanins [11,12,13] and anthocyanin-rich plant extracts [12,14,15] on the intestinal α-amylase in *in vitro* experiments, thus reducing starch degradation and glucose formation by α-glucosidase, have also been proposed. However, systematic studies of the impact of their B-ring substitution and the effect of an additional acylation do not exist. In addition, as inhibitor constants are strongly dependent on the assay conditions used, literature data are only comparable to a very limited extent. In the present study, the inhibitory effects of Plg, Cyd, Dpd, Peo, and Mlv-3-glucoside, as well as acylated Cyd-3-glycosides, isolated from commercial black carrot concentrate, were evaluated.

In order to compare the α-amylase inhibition of different anthocyanins with further phenolic structures, an enzyme activity assay, which was already applied in a previous study [16], was used. This assay is based on the conversion of 2-chloro-4-nitrophenyl-4-O-ß-galactopyranosyl maltoside (GalG_2_CNP), followed by UV/Vis spectroscopic detection of the released CNP. As anthocyanins and, in particular, acylated anthocyanins exhibit a strong self-absorption at the corresponding detection wavelength (405 nm), isothermal titration calorimetry (ITC) was used additionally for higher anthocyanin concentrations and the predominantly acylated structures obtained from black carrot. In contrast to the frequently used multiple injection experiment, which provides binding constants between the polyphenols and proteins [17], the simple and quick single injection experimental setup (enzyme addition to the substrate with or without the presence of an inhibitor and followed by reaction tracking) was used [18,19]. This screening approach provides comparable information to UV/Vis detection of the inhibitory effect of polyphenols.

## 2. Materials and Methods 

### 2.1. Materials, Solvents, and Reagents

A saline suspension of α-amylase from porcine pancreas (Lot #SLBN9170V, 42 mg/mL protein, 1151 units/mg (1 unit is equivalent to the release of 1.0 mg of maltose from starch in 3 min at pH 6.9, 20 °C)), 2-chloro-4-nitophenol (CNP), and 2-(N-morpholino)ethansulfonic acid (MES) were bought from Sigma Aldrich (Steinheim, Germany). The 2-chloro-4-nitrophenyl-4-O-ß-galactopyranosyl maltoside (GalG_2_CNP) was purchased from Sorachim (Lausanne, Switzerland) and acarbose (ACA) was purchased from Acros Organics (Geel, Belgium). Sodium hydroxide, and hydrochloric and formic acid were obtained from Grüssing (Filsum, Germany); sodium azide, and sodium acetate and chloride, as well as calcium chloride, from Roth (Karlsruhe, Germany); methanol and acetonitrile from Fisher Scientific (Loughborough, UK); and ethyl acetate from Sigma Aldrich (Steinheim, Germany). All reagents and solvents were of analytical grade. Reversed phase cartridges (CHROMABOND® Octadecyl modified silica gel, end capped; 1.000 and 10.000 mg) for solid phase extraction (SPE) were obtained from Macherey-Nagel (Düren, Germany). Anthocyanin-3-glucosides were obtained as chlorides from Phytolab (Vestenbergsreuth, Germany) and stored at −80 °C. Commercial black carrot concentrate, containing additional citric acid, was donated by Wild (Valencia, Spain) and stored at −20 °C. Ultrapure water (ELGA PurLab flex, Veolia Waters, Celle, Germany) was used throughout. 

MES^+^ buffer was prepared by mixing 50 mM MES, 10 mM sodium acetate, 41.5 mM sodium chloride, 5 mM calcium chloride, and 152 mM sodium azide. The pH was adjusted to 6.1 using 5 M NaOH. Anthocyanin stock solutions were prepared in 0.1% HCl. Concentrations of the stock solutions were determined by UV/Vis spectroscopy (Spectrostar® Nano, BMG Labtech, Ortenberg, Germany; quartz glass cuvette *d* = 1 cm, Helma Analytics, Müllheim, Germany) by the use of the following absorption coefficients ε (determined experimentally at λ_max_, results for the absorption coefficients determination can be found in Table A1): 24.500 L/(cm∙mol) for Plg-3-glc (511 nm), 25.500 L/(cm∙mol) for Cyd-3-glc (510 nm), 25.500 L/(cm∙mol) for Dpd-3-glc (516 nm), 26.000 L/(cm∙mol) for Peo-3-glc (497 nm), and 24.000 L/(cm∙mol) for Mlv-3-glc (519 nm). To avoid degradation, all stock solutions were prepared on the day of experiment.

### 2.2. Preparation of the Black Carrot Anthocyanin Extract (BC-ACY) by Solid Phase Extraction

The commercial black carrot concentrate was diluted with ultrapure water to 10% concentrate content, and solid phase extraction (SPE) was performed on a C18 cartridge, activated by methanol, and rinsed with water afterward. Cell wall components, sugars, and edible acids were eluted from the cartridge with 0.1% HCl and then non-anthocyanin phenolics by ethyl acetate. Subsequently, anthocyanins were eluted by the use of acidified methanol, (0.1% HCl). This eluate (BC-ACY) was concentrated by a rotary evaporator (28 °C; 10 mbar) several times to remove all traces of methanol and re-diluted with ultrapure water. 

The BC-ACY was analyzed on a 1260 Infinity HPLC equipped with a G1311B quadrupole pump, a G1364 C fraction collector, and a diode array detector (Agilent Technologies, Santa Clara, CA, USA). Separation was obtained on an Atlantis® T3 (250 × 4.6 mm; 5 μm; Waters Corp., Milford, MA, USA) column equipped with a pre column (EC 4/2 UNIVERSAL RP-precolumn, Macherey-Nagel, Düren, Germany) using a flow of 0.6 mL/min. Eluent A consisted of 30 mL/L acetonitrile and 100 mL/L formic acid in ultrapure water. Eluent B was acetonitrile acidified with 100 mL/L formic acid. The gradient started with 6% eluent B, which was raised to 50% within 30 min, followed by a rise to 100% and recondition. Anthocyanin composition was evaluated at 520 nm and the absence of non-anthocyanin phenolics was checked at 280, 320, and 370 nm.

### 2.3. Isolation of Black Carrot Anthocyanins by Preparative HPLC 

Preparative HPLC was also performed on a 1260 Infinity and separation was carried out with a SUPELCO Discovery® C18 569227-U (100 × 21.2 mm; 5 μm; Sigma-Aldrich, St. Louis, MO, USA) column operated at room temperature 25 ± 2 °C. The mobile phase consisted of 47 mL/L acetonitrile and 100 mL/L formic acid in water. The flow was 5 mL/min for 50 min and all fractions were collected manually based on the absorption at 520 nm. Respective fractions were pooled and the acetonitrile was removed on the rotary evaporator. For concentration purposes, an additional SPE was carried out on a C18 cartridge (1.000 mg). The purified anthocyanin structures were eluted with 0.01% hydrochloric acid in methanol. The anthocyanins were dried using a vacuum concentrator (RVC 2−18 CD plus, Martin Christ Gefriertrocknungsanlagen GmbH, Osterode, Germany), re-diluted with acidified water (0.01% HCl), and stored at −20 °C until use. 

### 2.4. Structure Characterization of the Black Carrot Anthocyanins by Mass Spectrometry

**Figure A1:**
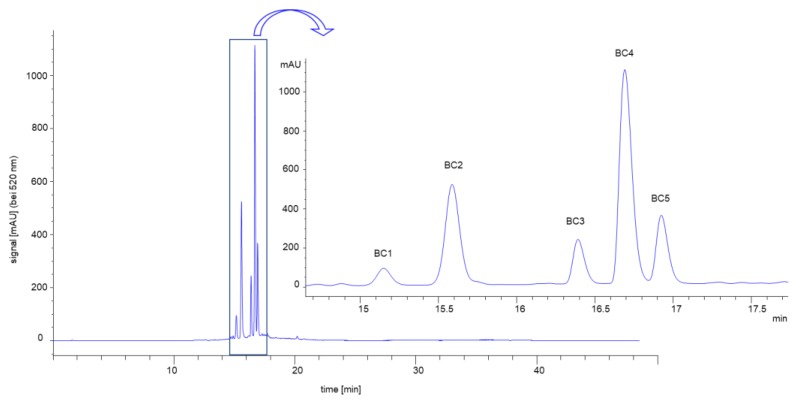
Chromatogram and respective structures of the complex black carrot anthocyanin extract (BC-ACY) at 520 nm. Abbr.: BC 1, Cyd-3-gal-xyl-glc; BC 2, Cyd-3-gal-xyl; BC 3, Cyd-3-gal-xyl-glc(sin); BC 4, Cyd-3-gal-xyl-glc(fer); BC 5, Cyd-3-gal-xyl-glc(cum).

Characterization of the isolated anthocyanin structures (for HPLC-chromatogram, see Figure A1) was performed with an AmaZon ETD (Bruker, Billerica, MA, USA) fitted with an ESI source. Analyses were carried out without previous chromatographic separation using an external syringe with a speed of 10 µL/min. Data acquisition and processing were performed with a Compass HyStar 4.1 and trapControl 8.0. Positive ion mass spectra were recorded in the range of m/z 200−2000 in the ultra-scan modus. Nitrogen was used as drying (8.5 L/min) and nebulizing (1.25 bar) gas. The nebulizer temperature was set at 200 °C and helium was used as collision gas at a pressure of 4 × 10^−6^ mbar. Data analyses were performed with DataAnalysis 4.4 (Bruker, Billerica, Ma, USA) (Table A2; Figure S2). 

### 2.5. Determination of the Absorption Coefficient of Black Carrot Anthocyanins Based on ^1^H-NMR

In order to avoid decay of the acylated structures during concentration and lyophilization and to avoid weighing errors due to low weights, absolute quantification of the black carrot anthocyanins was performed in solution by quantitative nuclear magnetic resonance spectroscopy (qNMR) at the Chemical and Veterinary Investigation Office (Chemisches Veterinär- und Untersuchungsamt, Karlsruhe, Germany). The measurement was carried out in 0.2 M KCl buffer adjusted to pH = 1.1 with 0.2 M HCl. The volumes of the 150 µL sample (in 0.01% HCl), 400 µL KCl buffer, and 50 µL deuterated water (D_2_O) were transferred to a 1.5 mL Eppendorf tube (Eppendorf AG) and mixed as required. The pH values ranged between 1.2 and 1.3 after 1 h equilibration. Then, 600 µL of the solution was transferred into 5 mm NMR tubes, and NMR spectra were recorded on a 400 MHz Bruker Avance (Bruker Biospin, Ettlingen, Germany) equipped with a BBI 400S1 H-BB-D-05 Z and an automatic sample changer (Sample Xpress). Proton spectra were acquired using the pulse program noesygppr1d_d7 (1D NMR spectra) with presaturation of the water signal and an additional d7 delay to keep the presaturation time only during d1. With a time domain (TD) of 128 k, 128 scans with 4 dummy scans were acquired, using a spectral width (SW) of 20.56 ppm (8223 Hz), acquisition time (AQ) of 7.97 s, and receiver gain (RG) of 32. Delay 1 (D1) was set to 4.00 s and delay 7 (D7) was set to 60.0 s. The temperature was set at 300 K (± 0.1 K). All spectra were automatically phased and baseline-corrected. NMR spectra were analyzed using TopSpin version 3.5 pl. 6 (Bruker Biospin, Ettlingen, Germany) and compound concentrations were determined using the pulse length-based concentration determination (PULCON) principle according to [20,21,22]. A ^1^H-NMR spectrum of a quantification reference solution (QuantRef), containing lactic acid and citric acid, was used to calculate the Electronic-Reference-to-access-*in-vivo*-Concentrations-(ERETIC) factor according to Equation (1).

(1) $$\mathrm{ERETIC}\;=\frac{I_{\mathrm{QRef}}\cdot \mathrm{SW}\cdot \mathrm{MW}}{\mathrm{SI}\cdot c_{\mathrm{QRef}}\cdot {\mathrm{NH}}_{\mathrm{QRef}}\cdot \mathrm{DF}}.$$

I_QRef_ = absolute integral of the considered signal from reference compounds
SW = spectral width
MW = molar weight (lactic acid: 90.080 g/mol; citric acid: 192.124 g/mol)
SI = size of the real spectrum after Fourier transformation
*c*_QRef_ = concentration of lactic/citric acid in the QuantRef standard (mmol/L).
NH_QRef_ = number of protons of lactic/citric acid causing the signal under consideration
DF = dilution factor

This factor was used to quantify the anthocyanins according to Equation (2).

(2) $${ß}_{x}=\frac{I_{x}\cdot \mathrm{SW}\cdot {\mathrm{MW}}_{x}\cdot P_{x}}{\mathrm{ERETIC}\cdot \mathrm{SI}\cdot {\mathrm{NH}}_{x}\cdot P_{\mathrm{QRef}}\cdot \mathrm{DF}}.$$

ß_x_ = mass concentration of the anthocyanin (mg/L).
I = absolute integral of the anthocyanin signal under consideration
SW = spectral width
MW_x_ = molar weight of the anthocyanin (g/mol).
P_x_/P_QRef_ = duration of the excitation pulse (µs).
SI = size of the real spectrum after Fourier transformation
NH_x_ = number of anthocyanin protons causing the signal under consideration
DF = dilution factor

Determination of the mass concentration ß was done in duplicate and calculated as an average for all aromatic protons. The absorptions of the NMR solutions were determined by UV/Vis spectroscopy (Lambda 25, Perkin Elmer, UV-Cuvette semi-micro cuvette *d* = 1 cm, Cat. No. 759.150 Brand, Wertheim, Germany), and the absorption coefficient ε (L/(cm∙mol)) was calculated according to Equation (3).

(3) $$\varepsilon \;=\frac{\mathrm{Abs}.\cdot \mathrm{DF}\cdot {\mathrm{MW}}_{x}}{{ß}_{x}\cdot l\cdot 1000}$$

Abs = absorption at λ_max_
MW_x_ = molar weight of the anthocyanin (g/mol)
ß_x_ = average mass concentration of the anthocyanin determined by qNMR (mg/L)
l = path length (1 cm)
DF = dilution factor
1000 = conversion to L

Determination of the absorption coefficient was performed for, at minimum, three different dilutions of, at minimum, two independent NMR solutions (Table A2). Detailed data, e.g., impacts of different proton signals used for concentration determination, are provided in the Appendix A (Table A3).

### 2.6. Enzyme Activity Assay

The enzyme activity assay, based on the hydrolytic cleavage of GalG_2_CNP by porcine intestinal α-amylase, was established by Homoki and co-workers [12] and already used by the authors to evaluate the inhibitory strength of different phenolic structures [16]. To enable the evaluation of the result obtained in the present study compared to the non-anthocyanin phenolics investigated previously, the assay conditions were identical. In short, to 12 different GalG_2_CNP concentrations ([S] = 0−2000 µM) in MES^+^ buffer, inhibitor [I] and 50 µL enzyme solution (c = 1.51 units/~100 pM, on the basis of supplier information) were mixed in a microtiter plate (Greiner BioOne, Frickenhausen, Germany; transparent, F-bottom). For blanks, the enzyme solution was substituted with MES^+^ buffer. The final volume per well was 250 µL. To quantify the reaction product, a linear calibration with eight different CNP concentrations in the range of 0−35 µM was performed for each plate. Dilutions of the anthocyanin stock solutions (0.1% HCl) were equilibrated at pH 6.1 for one hour. Final inhibitor concentrations were limited due to its strong absorption at 405 nm to 12.5, 25, and 50 µM. For each anthocyanin structure, the assay was conducted on two to three different days with freshly prepared stocks of substrate, enzyme, and inhibitor.

In addition to the Michaelis Menten constant (K_m_) and the maximal conversion rate (v_max_) values (Table S1), inhibition constants (K_ic_, K_iu_) were calculated by fitting the data by two approaches: (i) According to the mixed inhibition equation (Equation (4)) or competitive inhibition equation (Equation (5)), applying an optimal global fit containing data from all inhibitor concentrations including the control ([I] = 0 µM); and (ii) by an independent fit according to the Michaelis Menten equation (Equation (6)), providing the apparent values K_m_^app^ and V_max_^app^ (Table S2) for each inhibitor concentration, which were then used to calculate the inhibition constants (Equations (7) and (8)).

(4) $$v_{0}=\;\;\frac{v_{\max}}{\frac{K_{m}}{\left[S\right]}\;\cdot \;\left(1+\frac{\left[I\right]}{K_{\mathrm{ic}}}\right)+\left(1+\frac{\left[I\right]}{K_{\mathrm{iu}}}\right)},$$

(5) $$v_{0}=\frac{v_{\max}}{\frac{K_{m}}{\left[S\right]}\;\cdot \;\left(\;1+\frac{\left[I\right]}{K_{\mathrm{ic}}}\right)+1},$$

(6) $$v_{0}=\frac{v_{\max\;}\cdot \;\left[S\right]}{K_{m}+\left[S\right]},$$

(7) $$K_{\mathrm{ic}}=\frac{\frac{v_{\max}{}^{\mathrm{app}}}{K_{m}{}^{\mathrm{app}}}\;\cdot \;\left[I\right]}{\frac{v_{\max}}{K_{m}}-\frac{v_{\max}{}^{\mathrm{app}}}{K_{m}{}^{\mathrm{app}}}},$$

(8) $$K_{\mathrm{iu}}=\frac{v_{\max}{}^{\mathrm{app}}\;\cdot \;\left[I\right]}{v_{\max}-{\;v}_{\max}{}^{\mathrm{app}}}.$$

The IC_50_ values of the different anthocyanins were derived from the inhibition constants for different substrate concentrations by the Cheng−Prusoff equation (Equation (9)) [23].

(9) $${\mathrm{IC}}_{50}=\frac{\left(K_{m}+\left[S\right]\right)}{\frac{K_{m}}{K_{\mathrm{ic}}}+\frac{\left[S\right]}{K_{\mathrm{iu}}}}.$$

K_M_: Michaelis Menten constant (µM)
v_max_: Maximal conversion rate of the uninhibited reaction (µM/min)
v_0_: Observed conversion rate at given substrate and inhibitor concentration (µM/min)
[S]: Substrate concentration
[I]: Inhibitor concentration
K_ic_: Inhibitor constant, reflecting the competitive inhibition in the equation (µM)
K_iu_: Inhibitor constant, reflecting the uncompetitive inhibition in the equation (µM)
v_max_^app^: Calculated maximal conversion rate, when the Michaelis Menten equation is used on an inhibited curve (µM/min)
K_m_^app^: Calculated Michaelis Menten constant, when the Michaelis Menten equation is used on an inhibited curve (µM)
IC_50_: Half maximal inhibitory concentration (inhibitor concentration needed to achieve 50% inhibition; it is dependent on the substrate concentration) (µM)

For data analyses and calculations, Origin 2018b (OriginLab, software extension Enzyme Kinetics) was used. The Levenberg−Marquardt algorithm procedure was chosen for iteration. All assays were performed in two independent replicates at different dates. Values are expressed in arithmetic means ± average deviation (AD). The statistical significance and differences between groups were analyzed by one-way analysis of variance (ANOVA), followed by Tukey’s test. Differences were considered significant when *p* < 0.05. Data were processed using Microsoft Excel® (2010).

### 2.7. Isothermal Titration Calorimetry (ITC)

To evaluate the inhibitory strength of 100 µM anthocyanin-3-glucosides and black carrot anthocyanins, GalG_2_CNP conversion to CNP was monitored by the released heat. According to the previous study, ITC measurements were carried out in a single injection experiment set-up on a MICROCALTM PEAQ-ITC calorimeter (S/N MAL1188053, Malvern Panalytical Almelo, Netherlands) [16]. Following an equilibration time of 10 min, 1 µL α-amylase (1 µM in 50 mM MES^+^ buffer) was added to 0.95 mM of the substrate with or without 100 µM of the phenolic inhibitor in the cell (V_cell_ = 0.207 mL). The temperature was maintained at 37 °C and the sample was stirred at 750 rpm throughout. The feedback/gain mode was set to high. The resulting heat profile was transformed [19] and the conversion rate was plotted against the substrate concentration [S] by an origin routine template provided by Malvern® for Origin version 7.

## 3. Results

### 3.1. Determination of α-Amylase Inhibition by Anthocyanin-3-Glucosides Using UV/Vis-Spectroscopy

**Figure 3:**
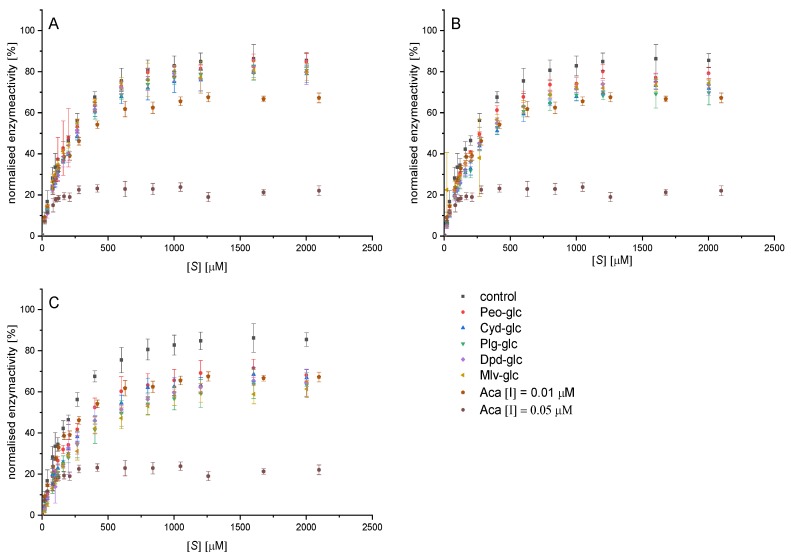
Decrease in conversion rate of 2-chloro-4-nitrophenyl-4-O-ß-galactopyranosyl maltoside (GalG_2_CNP) by addition of 12.5 (**A**), 25 (**B**), and 50 µM (**C**) anthocyanin-3-glucosides and 0.01 and 0.05 µM acarbose, normalized on the v_max_ value of the uninhibited reaction. Abbr.: [S], substrate concentration; Plg-3-glc, pelargonidin-3-glucoside; Cyd-3-glc, cyanidin-3-glucoside; Dpd-3-glc, delphinidin-3-glucoside; Peo-3-glc, peonidin-3-glucoside; Mlv-3-glc^#^, malvidin-3-glucoside; ACA^#^, acarbose; ^#^values already published by Kaeswurm et al. [16].

**Figure A2:**
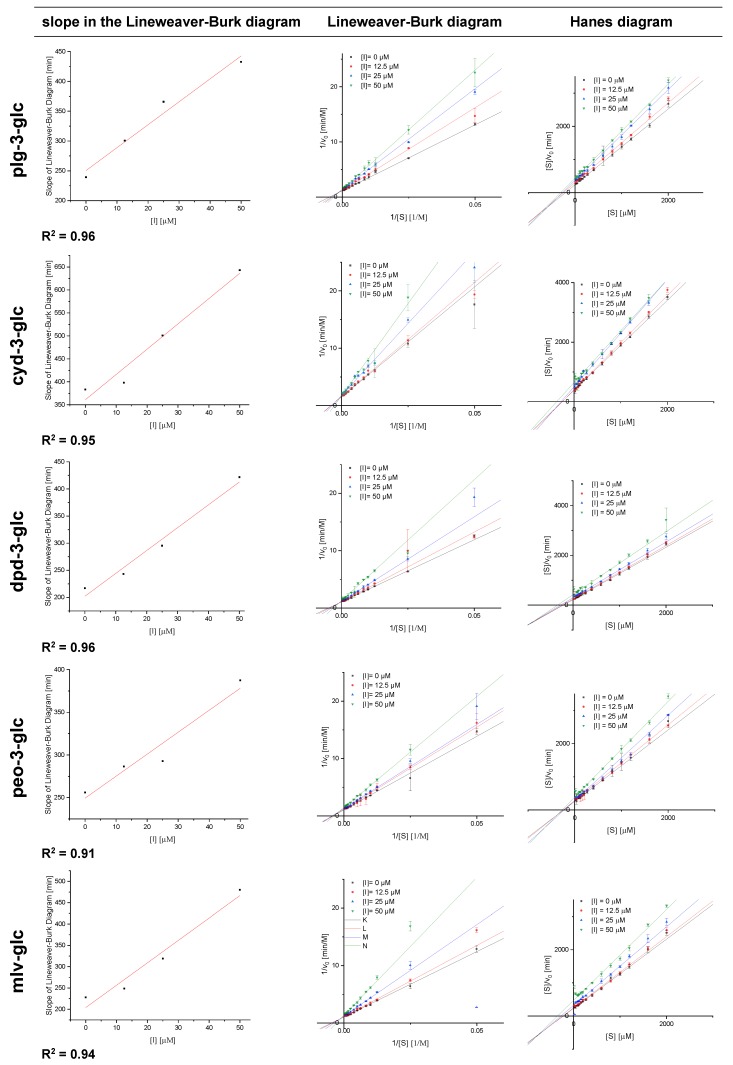
Linearized plots to demonstrate complete inhibition and to evaluate inhibition type. Abbr.: Plg-3-glc, pelargonidin-3-glucoside; Cyd-3-glc, cyanidin-3-glucoside; Dpd-3-glc, delphinidin-3-glucoside; Peo-3-glc, peonidin-3-glucoside; Mlv-3-glc, malvidin-3-glucoside; [I], inhibitor concentration; [S], substrate concentration; v_0_, measured conversion rate at used substrate and inhibitor concentration.

The normalised Michaelis−Menten diagrams (Figure 3) illustrate a significant inhibition of α-amylase by anthocyan-3-glucosides at a concentration of 25 and 50 µM compared to the control. However, the inhibitory effects of the individual anthocyanin structures do not differ from each other. All anthocyanin structures are reversible inhibitors as the plot of enzyme activity over time does not exhibit an exponential decrease in activity over time (exemplary data for cyd-3-glc provided in Figure S1) [24]. The slope in the Lineweaver−Burk diagram (1/v_0_ vs. 1/[S]) plotted versus the inhibitor concentration shows a linear trend, indicating a complete inhibition. Linearized plots such as Lineweaver−Burk and Hanes (Figure A2) point to a mixed competitive inhibition. However, due to contortion in the loadings for different substrate concentrations in these plots, conclusions about the inhibition types are not reliable. 

Fitting the curves according to the equation for the mixed inhibition (i, Equation (4)) and competitive inhibition equation (Equation (5)) with Origin enables the determination of the inhibition constants K_ic_ and K_iu_ (Table 1). Both values calculated by the apparent values of K_m_ and V_max_ (ii, Equations (7) and (8), Table S2) are in the same range. K_ic_, K_iu_, and their ratio do not differ significantly among the anthocyanidin-3-glucosides (Table 1). The lower R^2^ (Table A4) values when fitting the data according to a pure competitive inhibition (Equation (4)) indicate, for all structures investigated, a mixed inhibition (K_ic_ ˂ K_iu_) with a high competitive portion (K_ic_/K_iu_ 0.31−0.45 (i); 0.26−0.58 (ii)). This implies an interaction of the anthocyanin-3-glucosides with both the enzyme and the enzyme-substrate complex. Nevertheless, substrate binding to the enzyme hinders the binding of the inhibitor, and the competitive part of the inhibition dominates. Based on the IC_50_ values calculated from the inhibition constants according to the Cheng−Prussoff equation (Equation (9)), minor differences in the inhibitory strength are obvious (Plg-3-glc > Mlv-3-glc > Cyd-3-glc > Dpd-3-glc~Peo-3-glc, Table 2, Figure 3). Nevertheless, these differences are not statistically relevant.

### 3.2. Determination of α-Amylase Inhibition by Anthocyanin-3-Glucosides Using Isothermal Titration Calorimetry 

**Figure 4:**
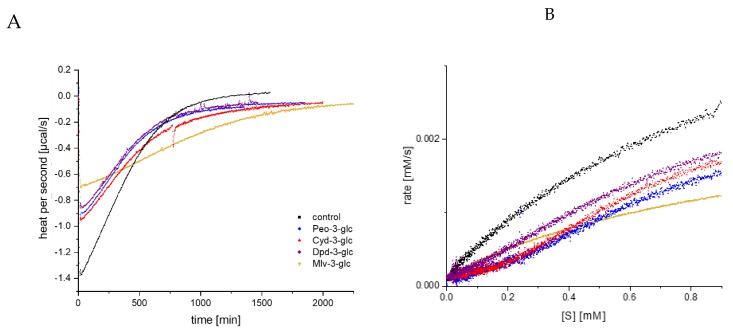
Thermogram (thermal power required to maintain constant temperature) for GalG_2_CNP conversion after addition of α-amylase in the presence of 100 µM anthocyanin-3-glc (**A**), and respective Michaelis Menten plot (**B**), converted from the thermogram (A). Abbr.: Cyd-3-glc, cyanidin-3-glucoside; Dpd-3-glc, delphinidin-3-glucoside; Peo-3-glc, peonidin-3-glucoside; Mlv-3-glc, malvidin-3-glucoside.

Due to the high self-absorption of anthocyanins at 405 nm, their effect on enzyme inhibition is impossible to distinguish by UV/Vis detection for concentrations above 50 µM. Therefore, according to our previous study [16], the inhibitory effect for 100 µM was additionally investigated by ITC. Substrate conversion was performed in the cell by a single enzyme addition in the presence or absence (control) of the inhibitor, and the thermal power to maintain a constant temperature (µcal/s), which is proportional to the reaction heat, was recorded over time [18,19,25]. The amplitude of the curve immediately after enzyme injection was reduced; the time span to return to the pre-injection base line, indicating the end of the reaction, was extended in the presence of the anthocyanin-3-glucosides (Figure 4A) [26]; and the thermogram was converted into a Michaelis Menten diagram (Figure 4B). Due to the higher concentration (100 µM), differences between the anthocyanin structures become more obvious. Nevertheless, the order of the inhibitory strength is identical to the lower concentrations detected by UV/Vis (Mlv-3-glc > Cyd-3-glc > Dpd-3-glc~Peo-3-glc).

### 3.3. Determination of α-Amylase Inhibition by Black Carrot Anthocyanins by UV/Vis-Spectroscopy

**Figure 5:**
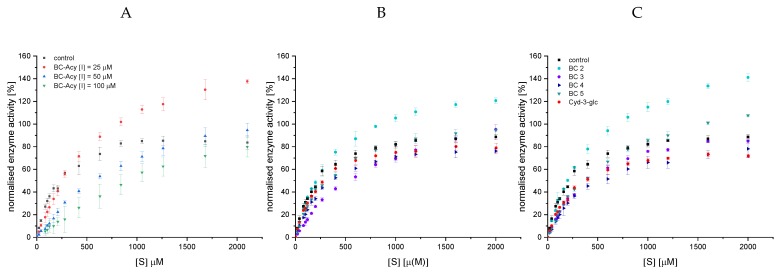
Decrease in conversion rate of GalG_2_CNP by addition of 25−100 µM black carrot anthocyanin extract (BC-ACY), the complex anthocyanin extract (**A**), and by 12.5 (**B**) and 25 µM (**C**) isolated anthocyanin-3-glycosides. Cyd-3-glc is included in the plot for comparison purpose. Abbr.: [S], substrate concentration; BC 2, Cyd-3-gal-xyl; BC 3, Cyd-3-gal-xyl-glc(sin); BC 4, Cyd-3-gal-xyl-glc(fer); BC 5, Cyd-3-gal-xyl-glc(cum).

Surprisingly, at higher substrate concentrations, the conversion rate (v_0_) in the presence of the complex anthocyanin mixture obtained by SPE (BC-ACY, ([I] = 25 µM, 50 µM)), containing five different anthocyanins (BC 1−5) (Figure A1), exceeded v_0_ observed in the control (Figure 5A). A v_0_ similar to or higher than the control was also detected for all anthocyanins isolated by preparative HPLC (Figure A1, Table A2) at 12.5 and 25 µM, except for Cyd-3-gal-xyl-glc(fer) (BC 4, Figure 5B,C). As this effect was present in both the complex anthocyanin extract and the individual structures isolated, it must be an artifact independent of the degree of purification. Up to now, we have been unable to explain this data properly. We assume that the presence of compounds not removed during the isolation process acts as co-pigments for CNP, enhancing absorption at 405 nm. This might simulate a higher product concentration and, thus, an increased conversion rate. We interpret the more pronounced effect for the non-acylated structures as a result of the reduced inhibition strength compared to the acylated structures. However, why the BC-ACY (Figure 5A), containing 60% BC 4, shows a much faster conversion is unexplainable, too.

**Figure A3:**
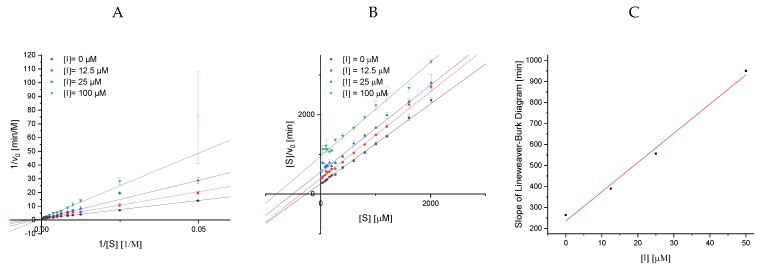
Linearized plots to evaluate inhibition type for Cyd-3-gal-xyl-glc(fer) (BC 4; A, B) and to demonstrate complete inhibition (**C**) (R2 = 0.99). Lineweaver−Burk plot (**A**); Hanes plot (**B**). Abbr.: [S], substrate concentration; [I], inhibitor concentration; v_0_, measured conversion rate at used substrate and inhibitor concentration.

Assuming that the improved conversion rate by the enzyme is identical for all anthocyanin structures, a minor inhibitory effect is found for BC 2, while BC 4 inhibits best. Unfortunately, due to the “faked” accelerated conversion rate, it is impossible to compare the black carrot anthocyanins in the UV/Vis-based enzyme activity assay to the non-acetylated anthocyanin-3-glucosides. Due to its high inhibition strength, the maximum conversion rate (v_max_) in experiments with BC 4 is always lower than the control. Therefore, despite improper determination of the conversion rate, analyses of the inhibitor type by linearization plots according to Lineweaver−Burk and Hanes (Figure A3) and the calculation of the inhibition constants are possible. Considering the errors, the intersection in the Lineweaver−Burk plot was on the ordinate (Figure A3A) and the lines in the Hanes diagram (Figure A3B) were parallel, particularly the inhibited samples. Therefore, both observations in combination with a linear increase in the Lineweaver−Burk slope with increasing inhibitor concentration (Figure A3C) indicate a complete competitive inhibition. 

**Figure 6:**
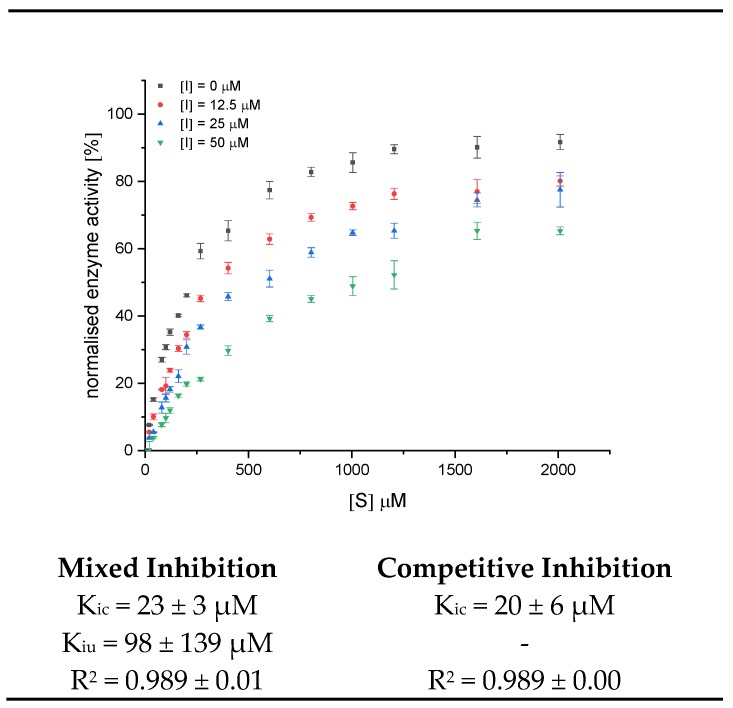
Decrease in conversion rate of GalG_2_CNP by addition of 12.5, 25, and 50 µM BC 4 (Cyd-gal-xyl-glc(fer)) and inhibition constants fitted according to the mixed and competitive inhibition. Abbr.: I, inhibitor; K_ic_, inhibition constant of the competitive part of the inhibition model; K_iu_, inhibition constant of the uncompetitive part of the inhibition model.

Inhibition constants for BC 4 were obtained by the global fit for the mixed and competitive inhibition according to Equations (4) and (5), respectively (Figure 6, Figure S3). The high standard deviation for α (calculated by origin, K_iu_ = K_ic_ ∙α) and a K_iu_ value markedly exceeding K_ic_ also support a predominantly competitive inhibition. The K_ic_ value of 20 µM should only be a rough estimation due to the general error (increase in conversion rate in the presence of BC 2, BC 3, and BC 5) in the UV/Vis detection. However, it indicates an improved inhibition compared to the non-acylated anthocyanin-3-glucosides (Table 1). 

### 3.4. Determination of α-Amylase Inhibition by Anthocyanin-3-Glucosides Using Isothermal Titration Calorimetry 

**Figure 7:**
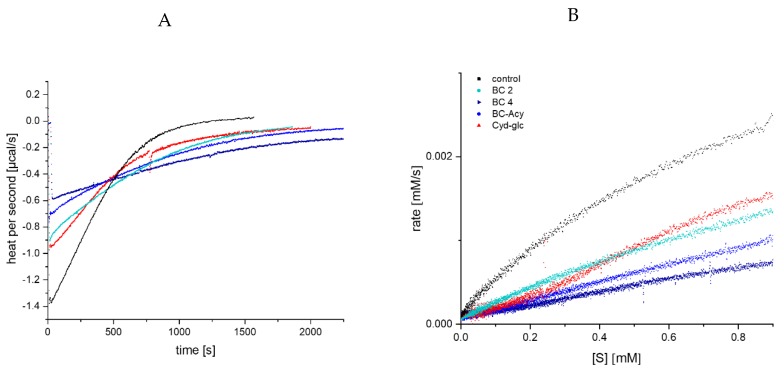
Thermogram for GalG_2_CNP conversion after addition of α-amylase in the presence of 100 µM black carrot anthocyanins (**A**), and respective Michaelis Menten plot (**B**), converted from (A). Abbr.: BC 2, Cyd-3-gal-xyl; BC 4, Cyd-3-gal-xyl-glc(fer); BC-ACY, the complex anthocyanin extract; Cyd-glc, Cyanidin-3-glucoside; [S], substrate concentration.

Due to the simulated increase in enzyme activity by addition of black carrot anthocyanins determined by UV/Vis spectroscopy, the inhibitory effect was additionally investigated by ITC to prove the assumed stronger inhibition of α-amylase by acylated structures than by non-acylated anthocyanins. The thermogram, as well as the extracted Michaelis Menten plot, shows a significantly stronger inhibition of BC 4 compared to the non-acylated BC 2 and Cyd-3-glc (Figure 7). For the complex anthocyanin extract, a significant improved inhibition is obvious, to a smaller extent than for BC 4, however. This finding is in contrast to the UV/Vis detection (Figure 5), which simulates an inhibition strength of the mixture equal to non-acylated black carrot anthocyanins. Despite the unapproachability of the inhibition constants (K_ic_, K_iu_), the ITC data demonstrate that the inhibition strength of acylated anthocyanins isolated from black carrot overtakes non-acylated compounds. Furthermore, the impact of a mono- or disaccharide seems to be negligible.

## 4. Discussion

Our data reveal that the substitution pattern on the anthocyanin B-ring seems to have little impact on α-amylase inhibition. Previous STD-NMR investigations of our group on the interaction epitope of various polyphenol structures point toward a major impact of the conjugated system [16]. Thus, inhibition strength is largely determined by the conjugated system elongation and, therefore, the interaction is presumably based on non-specific hydrophobic interactions. The results of the present study support this hypothesis as the conjugated system is not influenced by the number of hydroxyl and methoxyl groups on the B-ring. This is in contrast to the results obtained *in vitro* and *in silico* for flavonols and (iso)flavones by Piparo et al. [27]. For these flavonoids, a marked impact of hydroxyl groups on the inhibition strength was observed and an interaction based on specific hydrogen bonds is proposed. In addition, Homoki et al. and Sui et al. found a significantly improved inhibition of α-amylase by Cyd-3-glc than for Mlv-3-glc and Peo-3-glc (Cyd-3-glc IC_50_ 180 ± 20 µM, Mlv-3-glc IC_50_ 675 ± 73 µM [12]; Cyd-3-glc IC_50_ 24 ± 3 µM, Peo-3-glc IC_50_ 75 ± 7 µM [13]). However, these studies were performed with human salivary α-amylase or with starch, which might explain the differences to our results. In contrast to other studies, Wiese et al. suggested a hydrophobic interaction of Cyd-3-glc with proteins including porcine intestinal α-amylase and saliva based on data obtained by quenching tryptophan fluorescence and evaluating the secondary protein structure by circular dichroism [28].

The effect of the glycosidic side chain is discussed controversially in the literature. Our data based on ITC experiments suggest no significant difference (Figure 7) between Cyd-3-glc (reference compound) and Cyd-3-gal-xyl (isolated from black carrot), this is in agreement with the results of Homoki et al. (Cyd-3-glc IC_50_ 180 ± 20 µM; Cyd-3-rut IC_50_ 200 ± 24 µM) [12] and Sui et al. (Cyd-3-glc IC_50_ 24 ± 3 µM; Cyd-3-rut IC_50_ 31 ± 7 µM) [13].

Anthocyanin structures are pH-dependent. At acidic conditions, the red colored charged and planar flavylium cation is predominant. With increasing pH, the colorless, neutral, and non-planar hemiketal is formed by a nucleophilic attack of water on C-2 [29]. Structural changes such as acylation have an impact on this hydration equilibrium (characterized by the pK_H_ value); nevertheless, the pK_H_ values of the non-acylated anthocyanin-3-glycosides in aqueous solution are comparatively similar around 3 (Cyd-3-glc: 3.02 ± 0.06 [30,31]; Pel-3-glc: 2.98 ± 0.06 [32]; Dpd-3-glc 2.36 ± 0.05 [33]). Thus, at assay conditions (pH 6.1), the uncharged hemiketal, where the conjugated system is interrupted, is the major structure for all anthocyanin-3-glycosides. With the slightly altered stability under assay conditions (pH 6.1 and 37 °C, Figure S4), causing ring opening, the transformation to the *cis*-chalcone and final degradation to phenolic acids and phloroglucinaldehyde might cause the statistically irrelevant differences and explain why Mlv-3-glc seems to be the best inhibitor among the anthocyanin-3-glycosides [34].

The improved inhibitory strength of the acylated structure might be explained by the additional aromatic system of the acid and, therefore, stronger interaction with the enzyme [16]. Furthermore, stability under slightly acidic conditions is improved due to intermolecular π–π interactions and a steric shielding of C-2. As a result, the proportion of the charged and planar flavylium cation is enhanced at slightly acidic and neutral conditions (pK_H_ above 4.3), and, thus, decay is reduced. 

A competitive inhibition type has been characterized for Cyd-3-glc and Peo-3-glc by Sui et al. [13] and for Cyd-3-glc and Mlv-3-glc by Homoki et al. [12]. However, the data in our study indicate a mixed inhibition. Whereas Sui et al. used a dissimilar enzyme activity assay, based on corn starch conversion [13], our assay was analogous to Homoki et al. [12]; nevertheless, the latter used human α-amylase, which might explain the different inhibition type. Furthermore, an additional reason for this discrepancy might be found in the different setups. Whereas Homoki et al. [12] varied the concentration of the inhibitor at a constant substrate concentration, we varied the substrate concentration at four different inhibitor concentrations (0, 12.5, 25, and 50 µM). Furthermore, we fitted our data according to the mixed (Equation (4)) and competitive inhibition (Equation (5)), in contrast to Homoki and co-workers [12] who assessed the inhibition type exclusively based on a Lineweaver−Burk plot. Significant K_iu_ values (Table 1) and poorer coefficient quality for the competitive inhibition (Table A4, Figure S3) support a mixed inhibition type, but demonstrate a strong competitive proportion (K_ic_ ˂ K_iu_).

As anthocyanins are also promising glucosidase inhibitors [15], an increased inhibitory effect for acylated anthocyanins is conceivable. Based on plant extracts containing acylated anthocyanins, Matsui et al. propose that acetylated anthocyanins are better inhibitors for α-glucosidases than the non-acylated representatives [35].

## 5. Conclusions

Our data show a marked inhibition of porcine intestinal α-amylase by anthocyanin-3-glucosides, independent of the B-ring substitution, and the presence of mono- or diglycosides at position C-3. The UV/Vis-based enzyme activity assay was unfeasible for acylated anthocyanins isolated from black carrot. However, reaction monitoring by ITC indicates a markedly stronger inhibition of the α-amylase by the acylated structures compared to anthocyanin-3-glycosides. To the best of our knowledge, the effect of acylated anthocyanin structures on α-amylase activity has been studied for the first time, indicating that a diet with an increased proportion of vegetables containing acylated anthocyanins might be a successful approach to prevent hyperglycemia and diabetes mellitus type II.

## Figures and Tables

**Table 1:** Inhibition constants K_ic_ and K_iu_ for anthocyanin-3-glycosides calculated by two different approaches.

-	(i) Inhibition Constants Obtained by the Global Fit (Equation (4))			(ii) Independent Fit for V_max_/V_max_^app^ and K_m_ and K_m_^app^ (Equation (6), Michaelis Menten, Table S2) and Calculation of the Inhibition Constants (Equations (7) and (8))		
Inhibitor	K_ic_ (µM)	K_iu_ (µM)	K_ic_/K_iu_	K_ic_ (µM)	K_iu_ (µM)	K_ic_/K_iu_
Plg-glc	57 ± 15 ^a^	151 ± 59 ^a^	0.39 ± 0.05 ^a^	69 ± 1 ^a^	158 ± 53 ^a^	0.46 ± 0.13 ^a^
Cyd-glc	81 ± 10 ***^a^	184 ± 56 ***^a^	0.45 ± 0.08 ***^a^	134 *^a^	228 *^a^	0.56 *^a^
Dpd-glc	62 ± 25 ^a^	204 ± 68 ^a^	0.34 ± 0.24 ^a^	63 ± 19 ^a^	270 ± 33 ^a^	0.26 ± 0.13 ^a^
Peo-glc	109 ± 39 ^a^	258 ± 78 ^a^	0.42 ± 0.23 ^a^	118 ± 42 **^a^	254 ± 82 **^a^	0.43 ± 0.04 **^a^
Mlv-glc ^#^	44 ± 4 ^a^	143 ± 44 ^a^	0.32 ± 0.07 ^a^	69 ± 17 ^a^	147 ± 50 ^a^	0.58 ± 0.35 ^a^

Values expressed as means of duplicate ± average deviation. Significant differences in the same line are indicated by different letters (*p* < 0.05). Abbr.: Plg-3-glc, pelargonidin-3-glucoside; Cyd-3-glc, cyanidin-3-glucoside; Dpd-3-glc, delphinidin-3-glucoside; Peo-3-glc, peonidin-3-glucoside; Mlv-3-glc^#^, malvidin-3-glucoside; K_ic_, inhibition constant of the competitive portion in Equations (4) and (7); K_iu_, inhibition constant of the uncompetitive portion in Equations (4) and (8); * the value of one day was used exclusively, due to the extremely low K_m_ value for one experiment; ** due to the nonconforming K_ic_/K_iu_ values for some concentrations, values are based on one experiment; *** calculated with a fixed K_m_ value for one day; # values already published by Kaeswurm et al. [16].

**Table 2:** IC_50_ values for anthocyanin-3-glycosides calculated by Cheng−Prussoff equation (Equation (9)) with K_ic_ and K_iu_ values obtained by different approaches.

-	(i) K_ic_ and K_iu_ Obtained by the Global Fit (Equation (4))				(ii) Independent Fit for v_max_/v_max_^app^ and K_m_ and K_m_^app^ (Equation (6), Michaelis Menten) and Calculation of K_ic_ and K_iu_ by Equations (7) and (8)			
I	[S] µM				[S] µM			
**-**	**10**	**100**	**10^3^**	**10^6^**	**10**	**100**	**10^3^**	**10^6^**
Plg-glc	58 ± 15 ^a^	71 ± 20 ^a^	117 ± 41 ^ab^	151 ± 59 ^a^	71 ± 2 ^a^	84 ± 7 ^a^	127 ± 31 ^a^	158 ± 53 ^a^
Cyd-glc	83 ± 11 ***^a^	97 ± 15 ***^a^	147 ± 36 ***^ab^	184 ± 56 ***^a^	137 *^a^	153 *^a^	201 *^a^	228 *^a^
Dpd-glc	64 ± 25 ^a^	77 ± 25 ^a^	134 ± 1 ^ab^	203 ± 68 ^a^	65 ± 20 **^a^	83 ± 24 **^a^	167 ± 20 **^a^	270 ± 32 **^a^
Peo-glc	112 ± 40 ^a^	134 ± 46 ^a^	209 ± 65 ^b^	258 ± 78 ^a^	121 ± 42 ^a^	142 ± 49 ^a^	212 ± 69 ^a^	254 ± 82 ^a^
Mlv-glc ^#^	46 ± 4 ^a^	58 ± 7 ^a^	106 ± 24 ^a^	143 ± 44 ^a^	71 ± 16 ^a^	83 ± 10 ^a^	121 ± 21 ^a^	147 ± 50 ^a^

Values expressed as means of duplicate ± average deviation. Significant differences in the same line are indicated by different letters (*p* < 0.05); Abbr.: Plg-3-glc, pelargonidin-3-glucoside; Cyd-3-glc, cyanidin-3-glucoside; Dpd-3-glc, delphinidin-3-glucoside; Peo-3-glc, peonidin-3-glucoside; Mlv-3-glc^#^, malvidin-3-glucoside; K_ic_, inhibition constant of the competitive portion in Equations (4) and (7); K_iu_, inhibition constant of the uncompetitive portion in Equations (4) and (8); v_max_, maximum conversion rate; K_m_^app^, apparent Michaelis Menten constant; v_max_^app^, apparent maximum conversion rate; I, inhibitor; [S], substrate concentration; * the value of one day was used exclusively, due to the extremely low K_m_ value for one experiment; ** due to the nonconforming K_ic_/K_iu_ values, IC_50_ values are based on one experiment; *** calculated with a fixed K_m_ value for one day; # values already published by Kaeswurm et al. [16].

## Supplementary Materials

The following graphs and tables are available online at https://www.mdpi.com/2304-8158/9/3/367/s1. Supplementary 1: Values for v_max_ and K_m_ determined by two different methods. Supplementary 2: Values for K_ic_ and K_iu_ fit according to a Michaelis Menten fit at different inhibitor concentrations (Equations (7) and (8)). Supplementary 3: Normalized enzyme activity of Cyd-3-glc (● 12.5 µM; ▲25 µM) during the enzyme assay per minute based on the activity of the uninhibited reaction (■) to demonstrate the reversible inhibition type. Supplementary 4: Individual mass spectra of the isolated compounds BC 1 (**A**), BC 2 (**B**), BC 3 (**C**), and BC 4 (**D**), and the respective fragments. Supplementary 5: Anthocyanin decay during 60 min at room temperature and additional 20 min at 37 °C at 12.5 (**A**), 25 (**B**), and 50 µM (**C**) (determined by HPLC-DAD (Diode Array Detector) at 520 nm and normalized to t_0_ = 100%). Supplementary 6: Plots to fit the data according to the mixed (Equation (4)) and the pure competitive (Equation (5)) inhibition.

## Appendix A

**Table A1:** Determination of the absorption coefficients ε for the anthocyanin-3-glycosides (reference compounds).

Compound	c (µM)	λ_max_ (nm)	ε for Each Concentration at λ_max_ Mean ± SD (L/(cm · mol))	ε Mean ± SD (L/(cm · mol))	Value for ε Used (L/(cm · mol))	Reference Value
Plg-3-glc	9.79 15.66 19.58 39.16	497	23.017 ± 259 24.733 ± 227 24.209 ± 1302 25.709 ± 129	24.417 ± 1121	24.500	27.300 (L/cm · mol) in 1% aqueous HCl [36], 15.600 (L/cm · mol) in 0.2 M KCl [37,38]
Cyd-3-glc	12.75 20.39 25.49 50.99	510	23.111 ± 1825 24.598 ± 1457 26.465 ± 1180 27.279 ± 453	25.363 ± 1974	25.500	26.900 L/(cm · mol) in 0.2 M KCl [37]
Dpd-3-glc	11.58 18.53 23.16 46.32	516	25.999 ± 523 24.862 ± 1555 25.718 ± 296 25.495 ± 212	25.519 ± 484	25.500	29.000 L/(cm · mol) in pH 1 buffer (not stated what buffer was used (0.2 KCl) [39]
Peo-3-glc	8.40 13.44 16.80 33.60	511	26.829 ± 802 25.939 ± 550 26.511 ± 624 25.420 ± 573	26.175 ± 623	26.000	11.300 L/(cm · cm) in 0.1% metanolic HCl [40]
Mlv-3-glc	8.19 13.10 16.37 32.75	519	23.025 ± 2349 23.653 ± 1670 24.078 ± 743 25.496 ± 349	24.063 ± 1049	24.000	28.000 L/(cm · mol) in 0.1 mol/L HCl [38]

The absorption coefficient was determined at four different concentrations (0.1% HCl). Abbr.: Plg-3-glc, pelargonidin-3-glucoside; Cyd-3-glc, cyanidin-3-glucoside; Dpd-3-glc, delphinidin-3-glucoside; Peo-3-glc, peonidin-3-glucoside; Mlv-3-glc, malvidin-3-glucoside; ε, extinction coefficient; c, concentration.

**Table A2:** Major anthocyanin structures characterized by mass spectrometry in the complex anthocyanin extract of black carrot.

No.	m/z [MH]+ and Respective Fragments *	Structure	Assigned Anthocyanin Structure According to [7]	Proportion of Total Peak Area at 520 nm (%)	Absorption Coefficient ε at 520 nm, pH1 ** (M · cm) *
**BC 1**	**743** 581 450 287	**Cyd-trihex** Cyd-dihex Cyd-hex Cyd	Cyd-gal-xyl-glc	4.75	32.500
**BC 2**	**581** 449 287	**Cyd-dihex** Cyd-hex Cyd	Cyd-gal-xyl	27.06	32.000
**BC 3**	**949** 743 287	**Cyd-trihex(sin)** Cyd-trihex Cyd	Cyd-gal-xyl-glc(sin)	10.25	36.000
**BC 4**	**919** 581 287	**Cyd-trihex(fer)** Cyd-dihex Cyd	Cyd-gal-xyl-glc(fer)	42.35	31.000
**BC 5**	-	**Cyd-trihex(cum)**	Cyd-gal-xyl-glc(cum)	15.42	34.000
**-**	-	**BC-ACY**	-	sum	average
**-**	-	**-**	-	100	3250

For a rough estimation of the individual content in the extract, the proportion of each individual compound was calculated from the area of the corresponding peak at 520 nm (Figure A1) assuming related absorption coefficients for all anthocyanin structures; * For individual mass spectra of the isolated compounds BC 1 to 5, see Supplementary 4. ** Exact extinction coefficient, including standard deviations provided in Table A3.

**Table A3:** Assigned absorption coefficient ε at λ_max_ of black carrot anthocyanins based on the concentration ß determined by q-NMR.

-	Compound	λ_max_ (pH1)	ß (mg/L)^a^	Absorption Coefficient ε^a^ Mean ± SD	ß (mg/L)^b^	Absorption Coefficient ε^b^ Mean ± SD	ß (mg/L)^c^	Absorption Coefficient ε^c^ Mean ± SD	ß (mg/L)^d^	Absorption Coefficient ε^d^ Mean ± SD
***Non-acylated anthocyanins***					-	-	-	-	-	-
**BC 1**	A	-	53.3	30,152.8	-	-	-	-	-	-
	B	-	87.8	34,427.3	-	-	-	-	-	-
	C	-	89.7	32,900.0	-	-	-	-	-	-
	**average**	513	-	**32,493.4 ± 2166.1**	-	-	-	-	-	-
**BC 2**	A	-	355.7	31,297.4	-	-	-	-	-	-
	B	-	351.3	33,040.2	-	-	-	-	-	-
	**average**	514	-	**32,168.8 ± 1232.3**	-	-	-	-	-	-
***Acylated anthocyanins***					-	-	-	-	-	-
**BC 3**	A	-	127.0 ± 6.7	-	125.9	35,395.1	134.2	33,202.2	121.0	36,840.8
	B	-	122.3 ± 3.0	-	121.6	36,898.3	125.6	35,731.7	119.7	37,492.1
	-	-	-	-	-	36,146.7 ± 1062.9	-	34,467.0 ± 1788.6	-	37,166.5 ± 460.5
	**average**	528	-	**35,926.7 ± 1363.1**	-	-	-	-	-	-
**BC 4**	A	-	908.2 ± 11.7	-	908.2	32,119.5	919.9	31,711.9	896.5	32,537.8
	B	-	914.3 ± 20.8	-	912.7	30,296.2	935.8	29,548.4	894.3	30,919.7
	-	-	-	-	-	31,207.9 ± 1289.3	30,630.2	1529.8 ± 5.0	-	31,728.8 ± 1144.2
	**average**	526	-	**31,188.9 ± 549.5**	-	-	-	-	-	-
**BC 5**	A	-	250.4 ± 8.9	-	251.5	33,023.2	258.7	32,107.0	241.0	34,453.6
	B	-	233.9 ± 4.4	-	236.3	34,734.8	228.9	35,862.0	236.6	34,696.7
	-	-	-	-	-	33,879.0 ± 1210.3	-a	33,984.5 ± 2655.2	-	34,575.2 ± 171.9
	**average**	525	-	**34,146.2 ± 375.2**	-	-	-	-	-	-

^a^ average concentration determined by quantitative nuclear magnetic resonance spectroscopy (qNMR) and respective absorption coefficient. For acylated anthocyanins, concentrations differ slightly depending on the protons used for calculation, ^b^ concentration calculations based on all aromatic protons, ^c^ protons of the aromatic acid, and ^d^ protons of the aglycon. Abbr.: BC 1, Cyd-3-gal-xyl-glc; BC 2, Cyd-3-gal-xyl; BC 3, Cyd-3-gal-xyl-glc(sin); BC 4, Cyd-3-gal-xyl-glc(fer); BC 5, Cyd-3-gal-xyl-glc(cum); β, mass concentration; λ_max_, wavelength of the highest measured absorption.

**Table A4:** Coefficient of determination for the fits based on a mixed and pure competitive inhibition for anthocyanin-3-glycosides.

Inhibitor	R^2^ Mixed Inhibition According to Equation (4)	R^2^ Competitive Inhibition According to Equation (5)
Plg-3-glc	0.99 ± 0.00	0.98 ± 0.01
Cyd-3-glc	0.97 ± 0.02	0.96 ± 0.03
Dpd-3-glc	0.98 ± 0.00	0.97 ± 0.00
Peo-3-glc	0.98 ± 0.02	0.97 ± 0.02

Plg-3-glc, pelargonidin-3-glucoside; Cyd-3-glc, cyanidin-3-glucoside; Dpd-3-glc, delphinidin-3-glucoside, Peo-3-glc, peonidin-3-glucoside; Mlv-3-glc, malvidin-3-glucoside.
