# Supplementary material for: Impact of B-Ring Substitution and Acylation with Hydroxy Cinnamic Acids on the Inhibition of Porcine α-Amylase by Anthocyanin-3-Glycosides
Source: Foods. 2020 Mar 21;9(3):367. doi: 10.3390/foods9030367 (PMC7142633; doi:10.3390/foods9030367)
Supplement: Supplementary file 1 [file foods-09-00367-s001.pdf]

Supplemental Part:

Impact of B-ring substitution and acylation with hydroxy cinnamic acids on the inhibition of porcine a-amylase by anthocyanin-3-glycosides

Julia A.H. Kaeswurm[1], Lisa Könighofer[1], Melanie Hogg[1], Andreas Scharinger[2] and Maria Buchweitz[1,*]

[1] Institute of Biochemistry and Technical Biochemistry, Department of Food Chemistry, University of Stuttgart; Germany

[2] Chemisches und Veterinäruntersuchungsamt Karlsruhe, Weißenburger Str. 3, 76187 Karlsruhe, Germany

***** Correspondence: e-mail: maria.buchweitz@lc.uni-stuttgart.de; phone: +49-711/68569231

Supplementary 1: Values for v_{max} and K_m determined by two different methods

	**optimal general fit according mixed inhibition equation (i)**		**fit according Michaelis Menten equation for the control ([I]=0), (ii)**	
inhibitor	v_{max} [µM/min]	K_m [µM]	v_{max} [µM/min]	K_m [µM]
Plg-3-glc	0.70 ± 0.23	216 ± 9	0.70 ± 0.23	221 ± 13
Cyd-3-glc	0.87 ± 0.32	233 ± 5*	0.82 ± 0.23	242**
Dpd-3-glc	0.88 ± 0.12	214 ± 16	0.87 ± 0.11	214 ± 21
Peo-3-glc	0.80 ± 0.13	205 ± 11	0.79 ± 0.11	208 ± 9
Mlv-3-glc	0.79 ± 0.20	179 ± 6	0.79 ± 0.18	188 ± 1
average	0,81 ± 0,07	209 ± 20	0,79 ± 0,06	214 ± 20

*K_m value of one day was fixed to 230, ** only one value is sensible, the other was not integrated in the calculation of the average. Abbr.: Plg-3-glc, pelargonidin-3-glucoside; Cyd-3-glc, cyanidin-3-glucoside; Dpd-3-glc, delphinidin-3-glucoside, Peo-3-glc, peonidin-3-glucoside; Mlv-3-glc#, malvidin-3-glucoside; v_{max}, maximum velocity; K_m, Michaelis Menten constant, [I], Inhibitor concentration.

Supplementary 2: Values for K_{ic} and K_{iu} fit according a Michaelis Menten fit at different inhibitor concentrations (eq. 6a,b).

	c [µM]	v_{max}/v_{max}^{app} [µM/min]	K_m/K_m^{app} [µM]	K_{ic} [µM]	mean ± SD [µM]	K_{iu} [µM]	mean ± SD [µM]
**Plg-glc**	0	0,70 ± 0,23	221 ± 13				
	12,5	0,65 ± 0,23	235 ± 2	91 ± 22	**69 ± 19**	187 ± 64	**158 ± 31**
	25	0,58 ± 0,22	258 ± 13	62 ± 7		126 ± 30	
	50	0,53 ± 0,23	325 ± 42	55 ± 19		163 ± 64	
**Cyd-glc**	0	0,81 ± 0,23	242*				
	12,5	0,79 ± 0,23	244*	236*	**134 ± 90**	146*	**179 ± 62**
	25	0,74 ± 0,26	285*	64*		141*	
	50	0,69 ± 0,21	300*	103*		250*	
**Dpd-3-glc**	0	0,87± 0,11	214 ± 21				
	12,5	0,84 ± 0,10	247 ± 33	66 ± 14	**63 ± 3**	364 ± 13	**270 ± 82**
	25	0,79 ± 0,09	273 ± 52	63 ± 17		233 ± 25	
	50	0,69 ± 0,03	335 ± 129	60 ± 27		212 ± 87	
**Peo-glc**	0	0,79± 0,10	208 ± 9				
	12,5	0,77 ± 0,12	211 ± 15	180*	**133 ± 40**	360*	**290 ± 61**
	25	0,74 ± 0,14	242 ± 8	107 ± 15		250*	
	50	0,65 ± 0,05	256 ± 7	112 ± 45		262 ± 92	
**Mlv-3-gl**	0	0.79 ± 0.18	188 ± 1				
	12,5	0.74 ± 0.24	194 ± 22	139*	**83 ± 52**	84*	**131 ± 41**
	25	0.67 ± 0.16	216 ± 3	74 ± 1		150 ± 14	
	50	0.60 ± 0.20	337 ± 4	36 ± 6		159 ± 66	

* only the value of one measurement was used. Abbr.: Plg-3-glc, pelargonidin-3-glucoside; Cyd-3-glc, cyanidin-3-glucoside; Dpd-3-glc, del-phinidin-3-glucoside, Peo-3-glc, peonidin-3-glucoside; Mlv-3-glc#, malvidin-3-glucoside; v_{max}, maximum velocity; K_m, Michealis Menten constant, v_{max}^{app}, apparent maximum velocity (calculated maximum velocity in inhibited reaction); $K_m^{qpp,}$ apparent Michaelis Menten constant (calculated maximum velocity in inhibited reaction); K_{ic}, competitive inhibition constant; K_{iu}, uncompetitive inhibition constant; SD, Standard deviation.

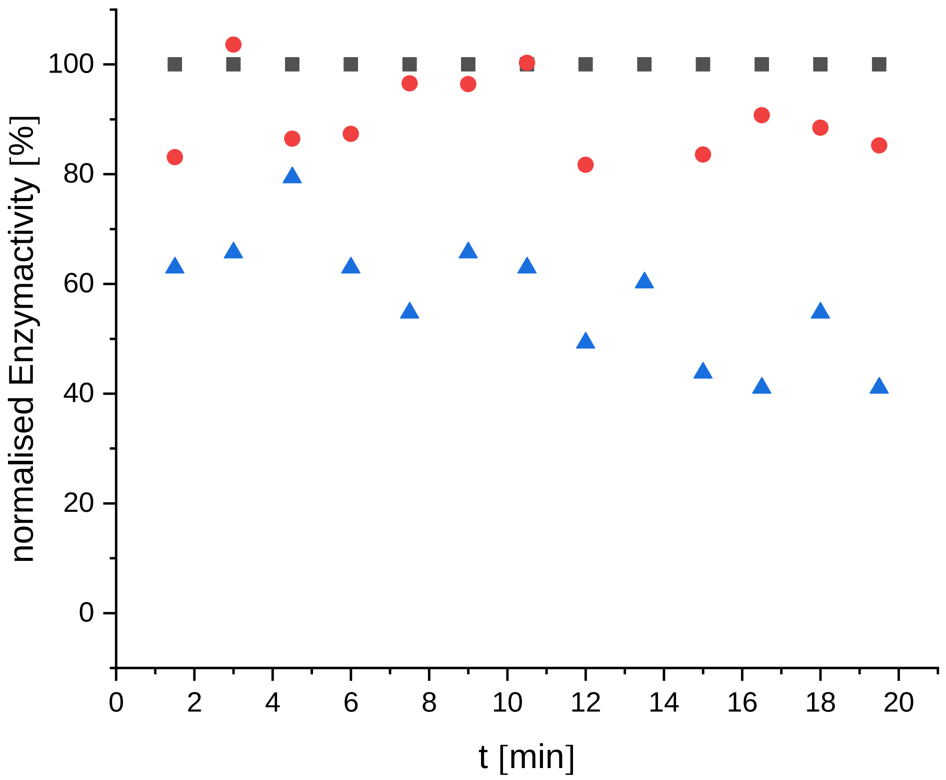

Supplementary 3: Normalised enzyme activity of Cydanidin-3-glucoside (● 12.5 µM; ▲25 µM) during the enzyme assay per minute based on the activity of the uninhibited reaction (■) to demonstrate the reversible inhibition type. Abbr.: t, time.

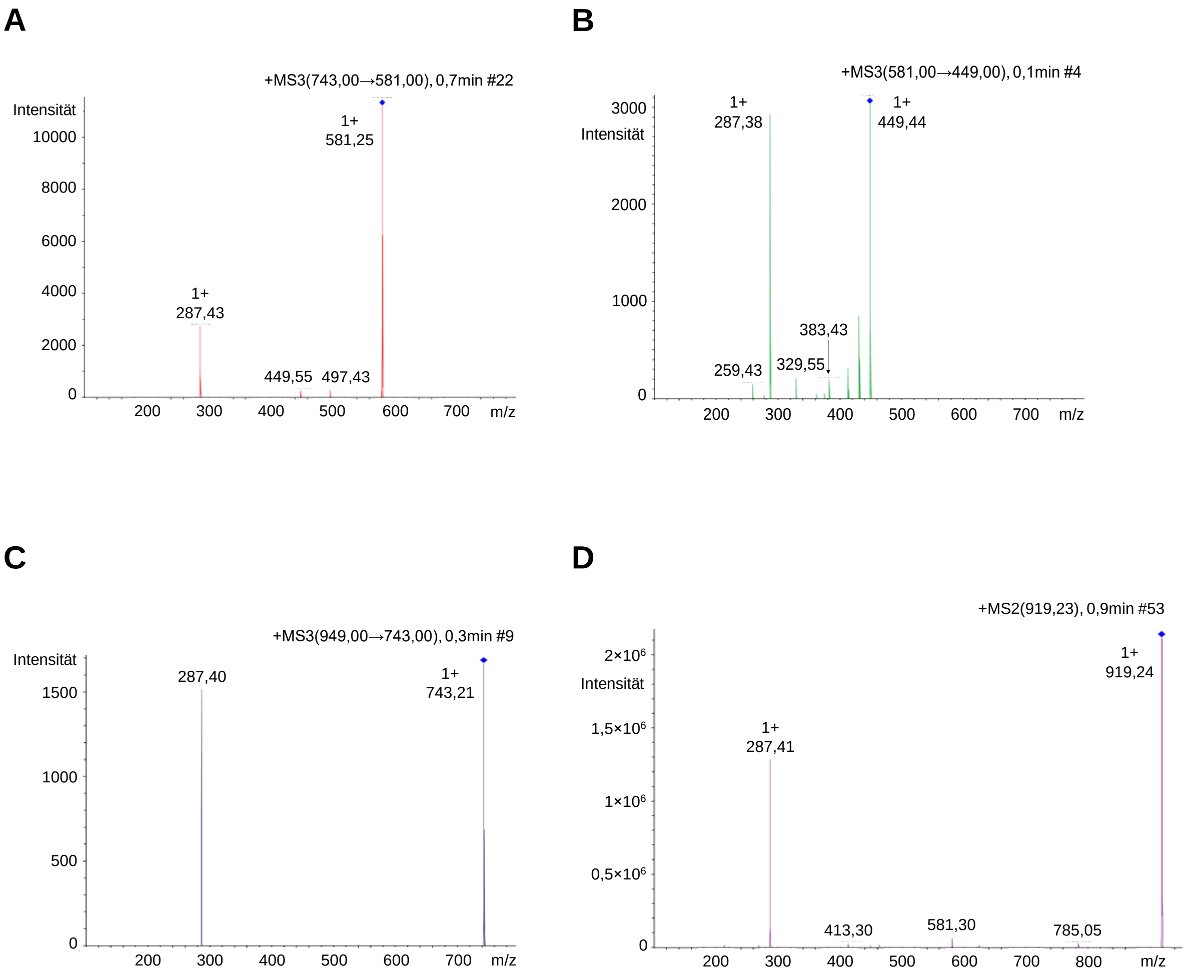

Supplementary 4: Individual mass spectra of the isolated compounds BC1 (A), BC2 (B), BC3 (C), BC4 (D) and respective fragments.

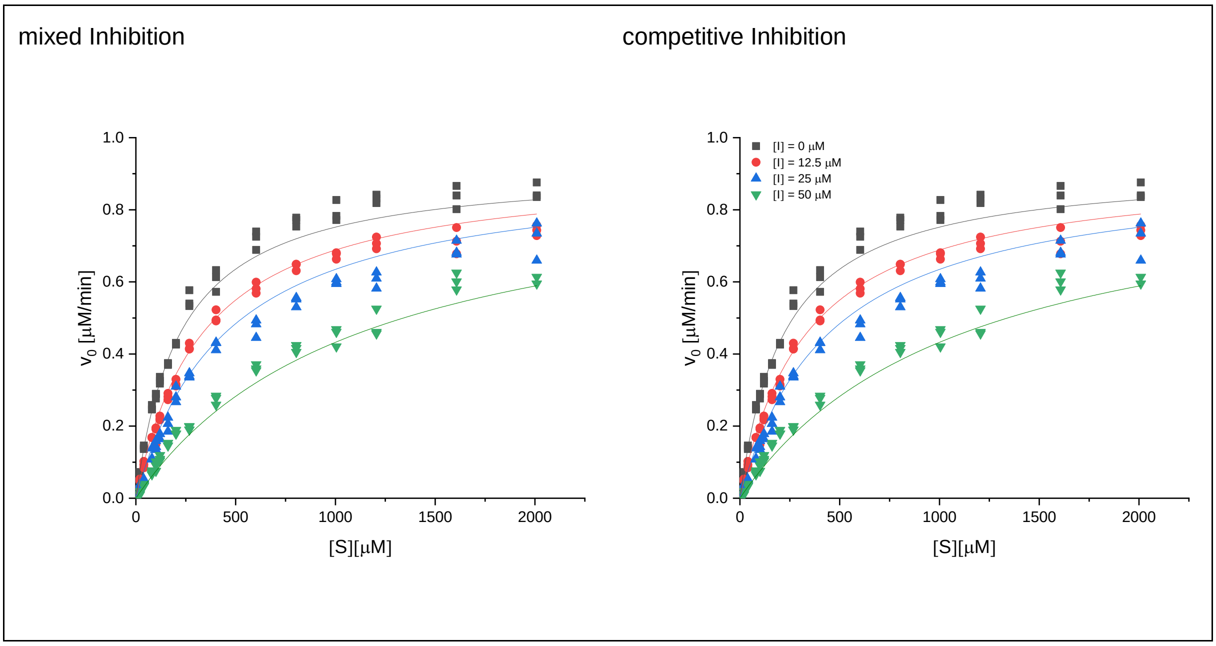

Supplementary 5: Plots to fit the data according the mixed (eq. 4a) and the pure competitive (eq. 4b) inhibition of. Cyd-3-gal-xyl-glc(fer).

To investigate anthocyanin stability during the enzyme activity assay, the assay preparation and the UV/Vis detection was mimicked.

The anthocyanin-3-glucosides were dissolved in 0.1% HCl (stock solution) and concentration was determined at 520 nm by UV/Vis spectroscopy as already described in the A1 . The stock solution was diluted with Mes+ buffer to 250, 125 and 62.5 µM corresponding to the inhibitor solutions used. Two samples each (200 µL) were taken after 0 and 60 min, diluted with 200 µL 0.1% HCl and anthocyanin-3-glucosides were quantitated relatively using the HPLC method described for black carrot anthocyanin extract in the main article. The areas for the samples taken at time 0 were set to 100%. After 60 min the Inhibitor solutions were further diluted with Mes+ 1:5 on the micotiter plate according to the inhibitor addition during the assay (analogous to the inhibitor concentrations of 50, 25 and 12.5 µM used in the assay). The microtiter plate was submitted to the plate reader and the protocol for the enzyme activity assay was started (20 min, 37°C, orbital shaking done in darkness). After 20 min samples of 200 µL were re-acidified with a similar volume of 0.1% HCl and anthocyanins were quantitated relatively to time zero by HPLC.

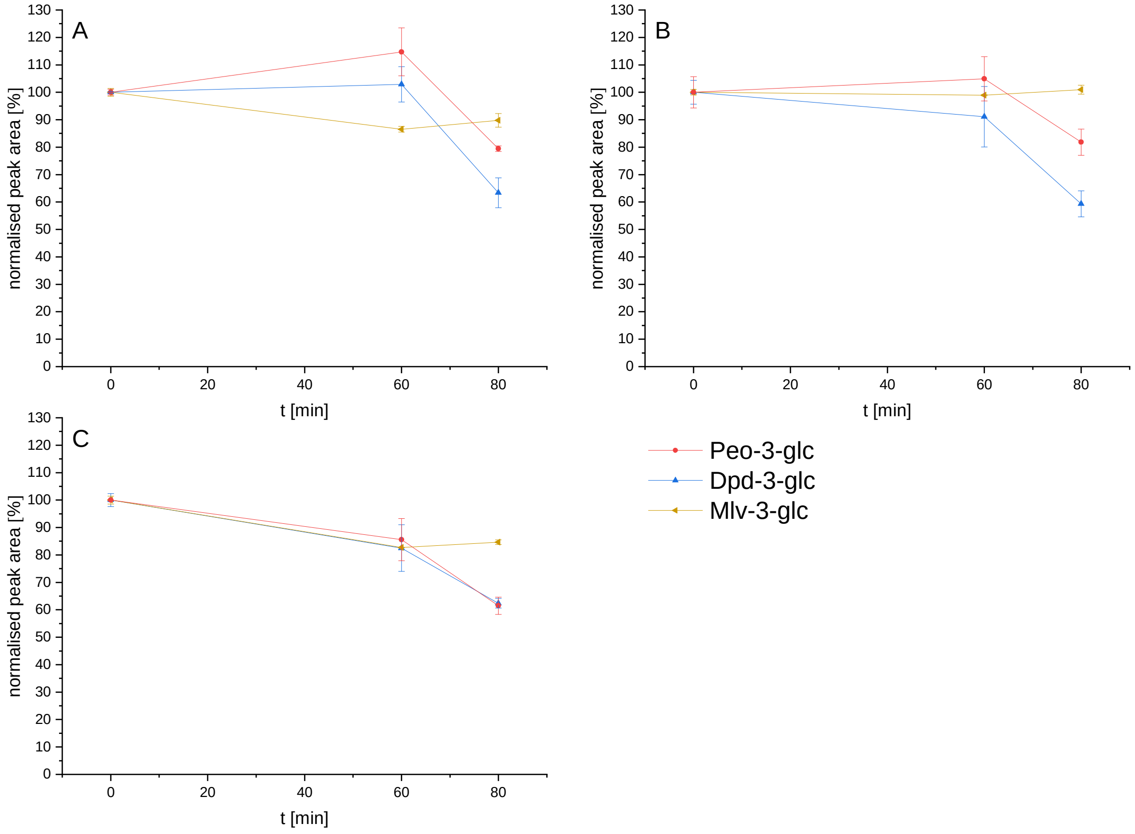

Supplementary 6: Anthocyanin decay during 60 min at room temperature and additional 20 min at 37°C at 12.5 (A), 25 (B) and 50 µM (C) (determined by HPLC-DAD at 520 nm and normalised to t_0=100%).Abbr.: Peo-3-glc; Peonidin-3-glucoside; Dpd-3-glc, Delphinidin-3-glucosid; Mlv-3-glc, Malividin-3-glucosid; t, time.
